# Anticarcinogenic activity of *Muntingia calabura* leaves methanol extract against the azoxymethane-induced colon cancer in rats involved modulation of the colonic antioxidant system partly by flavonoids

**DOI:** 10.1080/13880209.2017.1371769

**Published:** 2017-09-05

**Authors:** Nur Liana Md Nasir, Noorsyaza Eddrina Kamsani, Norhafizah Mohtarrudin, Fezah Othman, Siti Farah Md. Tohid, Zainul Amiruddin Zakaria

**Affiliations:** a Department of Biomedical Science, Faculty of Medicine and Health Sciences, Universiti Putra Malaysia, Serdang, Selangor, Malaysia;; b Department of Pathology, Faculty of Medicine and Health Sciences, Universiti Putra Malaysia, Serdang, Selangor, Malaysia;; c Halal Product Research Institute, Universiti Putra Malaysia, Serdang, Selangor, Malaysia

**Keywords:** Chemoprevention, endogenous antioxidant system, rutin

## Abstract

**Context:** Leaves of *Muntingia calabura* (Elaeocarpaceae) are widely used in traditional medical practice; scientific findings show various pharmacological activities. However, its anticancer effect has not been investigated thoroughly yet.

**Objective:** The objective of this study is to study the chemoprevention effects of MEMC_L_ against azoxymethane (AOM)-induced colon cancer and to examine the involvement of endogenous antioxidants

**Materials and methods:** Male Sprague–Dawley rats, divided into five groups (*n* = 7), were injected intraperitoneally once weekly for 2 weeks with 15 mg/kg AOM, except for the normal group (received saline). The animals were then administered orally for 8 weeks with 8% Tween-80 (vehicle; normal group), 8% Tween-80 (vehicle; cancer group) or, 50, 250 or 500 mg/kg MEMC. After treatments, colon samples were collected from each rat for the histopathological analysis, quantification of aberrant crypt foci formed and determination of colon antioxidant levels. MEMC was also subjected to HPLC analysis.

**Results:** The extract exerted significant (*p* < 0.05): (i) anti-carcinogenesis activity, indicated by a decrease in the total aberrant crypt formation; (ii) antioxidant activity by increasing the colon tissue antioxidant markers [i.e., superoxide dismutase (SOD), catalase (CAT) and glutathione (GSH)] and reducing the oxidant marker (i.e., malonaldehyde (MDA) levels in comparison with the cancer group. HPLC analysis demonstrated the presence of rutin.

**Discussion and conclusions:** *Muntingia calabura* leaves exert anticancer effect against AOM-induced colon cancer possibly via the action of flavonoids on the colon tissue antioxidant activity.

## Introduction

Colon cancer is recognized as the third most common cancer worldwide with high morbidity and mortality, and the fourth common cause of death (Haggar and Boushey 2009). The need to find alternative and new anticolon cancer agents was further triggered by the fact that most of the available therapeutic treatments, such as radiotherapy, chemotherapy and surgery, are afflicted with severe side effects (i.e., hair loss, immunosuppression, diarrhoea and bleeding) (Kranz and Dobbelstein 2012). Thus, there is an urgent interest and demand in using plant phytoconstituents to identify novel chemotherapeutic agents that are more effective and have minimal adverse side effects.

One of the carcinogenic agents commonly use in colon cancer study is azoxymethane (AOM), which works via the modulation of mechanism that is oxidative stress dependent (Waly et al. 2012). The ability of AOM to act as a procarcinogen in colon carcinogenicity is attributed to the formation of ultimate carcinogenic metabolite, the highly reactive methyldiazonium (MD) ion, which is responsible for lipid peroxidation that leads to oxidative stress and decrease in the total antioxidants capacity of colonic cells (Al-Numair et al. 2011; Hamiza et al. 2012). Specifically, AOM mediated the reduction of glutathione (GSH) leading to impairment in the total antioxidant capacity of rats colonic cells (Waly et al. 2012). In addition, MD will cause alkylation of macromolecules in the liver and colon, as well as the addition of the methyl group at the O6 position of guanine. This will cause point mutation of (G:C) to (A:T) that caused changes from glycine to aspartic acid (Vogelstein et al. 1988). Therefore, it is plausible to suggest that any compounds that are capable of attenuating oxidative stress might also have the ability to exert anticancer activity.

One of the plants that is postulated to possess anticancer activity is *Muntingia calabura* L. (Elaeocarpaceae). Throughout the world, *M. calabura* is well known as Jamaican cherry and, in Malaysia, it is locally known as ‘kerukup siam’ or ‘buah ceri’. *Muntingia calabura* has a long history of native folk medicine among the peoples of Peru, Cambodia, Columbia, Vietnam and the Philippines (Mahmood et al. 2014) and scientific studies carried out on the leaves, in particular, have demonstrated various medicinal activities such as cytotoxic, antiproliferative, anti-inflammatory and antioxidant (Mahmood et al. 2014; Zakaria et al. 2014; Rofiee et al. 2015). Earlier cytotoxic study revealed the potential of several flavonoids (i.e., (2S)-5′-hydroxy-7,3′,4′-trimethoxyflavanone and 4′-hydroxy-7-methoxyflavanone) and chalcones (i.e., 2′,4′-dihydroxychalcone and 2′,4′-dihydroxy-3′-methoxychalcone) isolated from the leaves of *M. calabura* to inhibit the growth of HT-29 (colon cancer) cell lines (Mahmood et al. 2014). Taking all these facts into account, the present study was performed to determine the anti-colon cancer activity of methanol extract of *M. calabura* leaves (MEMC_L_) against the AOM-induced colon cancer model in rats.

## Materials and methods

### Collection of plant materials and the preparation of MEMC_L_

The leaves of *M. calabura* were collected around Universiti Putra Malaysia (UPM), between July and December 2012. A voucher specimen (SK 2200/13) was identified by a botanist from the Institute of Bioscience, Universiti Putra Malaysia (UPM) by comparison with specimens available at the Herbarium of the Laboratory of Natural Products, Institute of Bioscience, UPM. The matured and dried leaves were powdered using a grinder into coarse powder. Briefly, 1 kg coarse powder of *M. calabura* was macerated within methanol (1:20, w/v) for 72 h in room temperature and this procedure was carried out three times using the same residue. The collected supernatant was pooled together and then evaporated using a rotary evaporator under reduced pressure.

### Experimental animal

The rats were kept in the standard condition of 12 h dark/light in the animal house of the Faculty of Medicine & Health Sciences, UPM. The animals were allowed free access to tap water and fed with standard pellet diet *ad libitium*. The study protocol of the present study was approved by the International Animal Care and Use Committee, Faculty of Medicine and Health Sciences, UPM (IACUC Ethical approval no. UPM/FPSK/PADS/BR-UUH/00488) and was conducted in accordance with international laws and policies (McPherson 1980). Prior to the experimentation, the rats were divided into five groups (*n* = 7).

### Anticarcinogenic study

#### Administration of carcinogen and MEMC_L_

After 2 weeks of acclimatization, all animals received carcinogen injection (AOM; Sigma Chemical Co., St. Louis, MO) intraperitoneally at the dose of 15 mg/kg once weekly for 2 weeks except for the normal control group, which received normal saline (Group 1) (Ghafar et al. 2012).

After the last administration of AOM, the rats in Groups 2, 3, 4 and 5 were treated with 8% Tween-80 (vehicle; cancer control group) or MEMC, at the doses of 50, 250 or 500 mg/kg, for 8 weeks in the volume of 10 mL/kg of body weight. On the contrary, the normal control group (Group 1) received normal saline. The body weight and the food intake of all rats were recorded weekly for the whole duration of the experiment. Each group and the respective treatment given are illustrated below:

**Table:** 

Groups		Administration
Control	I (Normal control)	Normal saline + normal saline
	II (AOM control)	AOM + 8% Tween-80
Treatment	III	AOM + 50 mg/kg MEMC
	IV	AOM + 250 mg/kg MEMC
	V	AOM + 500 mg/kg MEMC

### Colon sample collection

After 8 weeks of treatment, rats from each group were sacrificed to allow quantification of aberrant crypt foci. Colon was collected and opened longitudinally before washed with normal saline. The opened colon was separated into two halves of equal width. Half of the colon was pinned on a board with flat mucosal on the top and fixed in 10% formalin overnight. The tissue was subjected to haematoxylin and eosin (H&E) staining. The remaining of the colon tissues were homogenized for antioxidant study.

### Histopathological examination and ACF counting

Collected colon tissues were fixed in 10% formalin and each part of colon samples were examined and counted for the total of ACF formation. Generally, ACF can be distinguished from normal crypts based on the morphological crypt seen under microscope: (1) darker in stain; (2) the size is enlarged and elongated; (3) thick epithelial lining; and (4) often had oval or slit-like lumen.

### Measurement of enzymatic activities of AOM-treated colon tissue homogenate

Approximately 100 mg of each colon tissue was homogenized in 1 mL of phosphate buffered saline (PBS) using a teflon homogenizer (Polytron, Heidolph, Germany). The tissue homogenate was then centrifuged at 4000 rpm at 4 °C for 25 min. The supernatant was collected and used for further assessment of enzymatic activities. The levels of superoxide dismutase (SOD), catalase (CAT), and glutathione (GSH) were determined using commercial kits (Cayman Chemical, Ann Arbor, MI), according to the instructions from the vendor. Additionally, the determination of the levels of malondialdehyde (MDA) was also carried out using commercial kits (Cayman Chemical, Ann Arbor, MI), based on the protocols provided by the manufacturer.

### Identification of flavonoids in MEMC_L_

Briefly, MEMC_L_, dissolved in methanol, was injected into a HPLC system consisting of the Waters Delta 600 with 600 Controller, photodiode array detector (Waters 996) (Milford, MA) and a Phenomenex Luna column (5 µm; 4.6 mm i.d. × 250 mm) (Torrance, CA). Two eluents labelled as A and B, which consist of the respective 0.1% aqueous formic acid and acetonitrile, were used to elute the phytoconstituents. The eluents were injected in the volume of 10 µL under the flow rate of 1.0 mL/min and column oven temperature set at 27 °C. The eluent was monitored at 366 nm. The retention time and UV spectra of major peaks were analyzed. MEMC_L_ was then spiked with several standard pure flavonoids in order to determine their presence in the extract (Zakaria et al. 2014).

### UHPLC-ESI-MS analysis of MMMC at the ratio of 1:1 (v/v)

The UHPLC-ESI-MS system consisted of Dionex Ultimate 3000 series including a binary pump with a built in solvent degasser, a diode-array detector, an autosampler equipped with a column oven and a column compartment (Thermo Fisher Scientific, San Jose, CA). The MMMC was separated on a Cortecs C18 column (1.6 µL, 2.1 × 50 mm I.D.; Waters Co., Milford, MA) maintained at 40 °C. The mobile phase consisted of a mixture 0.1% formic acid in water and a mixture 0.1% formic acid in acetonitrile. A constant flow of 0.3 mL/min was applied. The acetonitrile percentages were the following: 0–5 min, 20%; 5–17 min, linearly from 20% to 60%; 17–20 min, 90%; 20–22 min, linearly from 90% to 5%; 22–30 min (re-equilibration step), 5%. The effluent from the chromatographic column was injected (10 µL) into a linear Q Exactive ion-trap-Orbitrap mass spectrometer (Thermo Fisher Scientific, San Jose, CA) equipped with an electrospray ionization (ESI) interface in the negative ion mode. The mass recognization was performed in a range of 150–1500 *m*/*z*. The main mass conditions were the followings: capillary temperature 320 °C, source voltage 3.2 kV, sheath gas (35 arbitrary units), auxiliary gas (15 arbitrary unit) and sweep gas (10 arbitrary unit). Nitrogen (>99.98%) was employed as sheath gas, auxiliary and sweep gas. Instrument control and data acquisition were performed with Chameleon 6.8 software and Xcalibur 2.2 software (Thermo Fisher Scientific, San Jose, CA).

### Statistical analysis

All data are presented as mean ± standard error of mean (SEM). Analyses of data were performed using the one-way analysis of variance (ANOVA) followed by the Dunnet *post hoc* test using the GraphPad Prism statistical software (version 5, GraphPad Software, La Jolla, CA). The *p* < 0.05 was set as the limit of significance.

## Results

### Effect of MEMC_L_ on body weight and relative organ weight of carcinogen-treated rats

**Figure 1.**
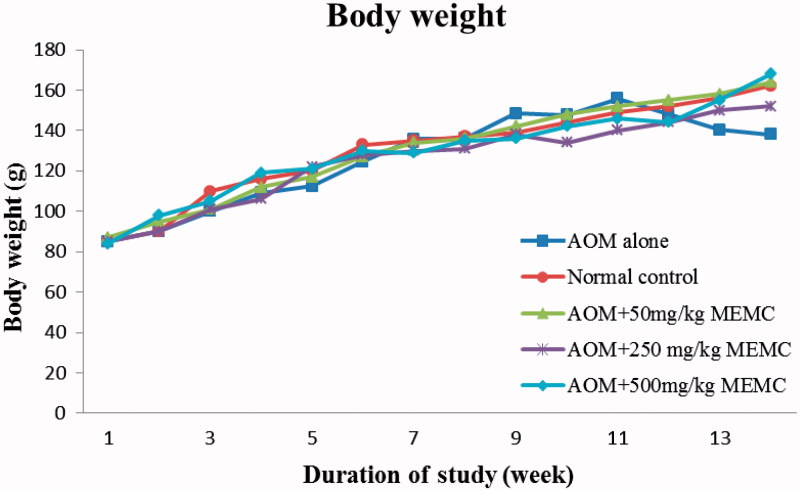
Body weight for male Sprague–Dawley rats for colon cancer chemopreventive study.

The change in body weight throughout the study is summarized in Figure 1. In general, all rats in Groups 1, 3, 4 and 5 showed an increase in the body weight except for Group 2 (cancer group), which showed a decrease in the body weight at the end of the experiment. However, no significant different (*p* > 0.05) observed in the relative organ/body weight ratio among the tested groups (Table 1).

**Figure 2.**
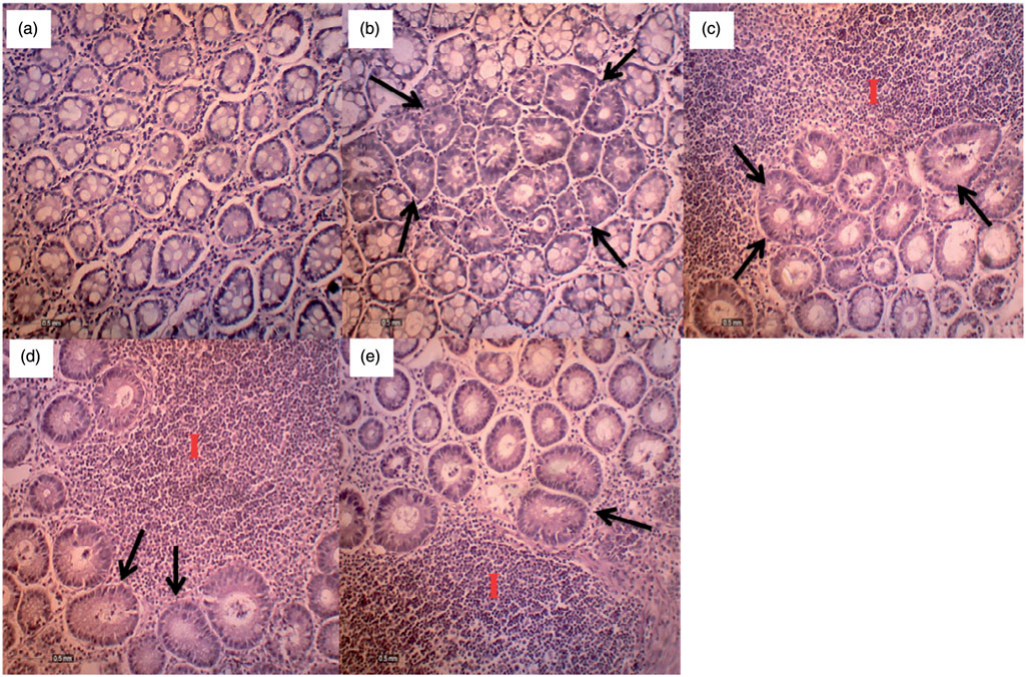
(a) Normal histoarchitecture of rat colon; (b) AOM control group; (c) section of colon tissue treated with AOM + 50 mg/kg MEMC; (d) section of colon tissue treated with AOM + 250 mg/kg MEMC; (e) section of colon tissue treated with AOM + 500 mg/kg MEMC (I – Infiltration of inflammatory cells. Black arrow – Aberrant crypt foci) (100 × magnification).

**Table 1.** Effect of MEMC on relative organ weigh (ROW) in AOM-treated rats.

% Organ weight/body weight	Normal control (normal saline alone)	Positive control (AOM alone)	AOM + 50mg/kg MEMC	AOM + 250mg/kg MEMC	AOM + 500 mg/kg MEMC
Spleen	0.232 ± 0.0398	0.257 ± 0.0296	0.275 ± 0.0161	0.295 ± 0.0361	0.347 ± 0.0186*
Heart	0.434 ± 0.0376	0.409 ± 0.0204	0.425 ± 0.0189	0356 ± 0.0224	0.434 ± 0.0328
Liver	2.654 ± 0.2637	2.951 ± 0.2124	3.037 ± 0.1840	2.868 ± 0.2441	3.520 ± 0.3584
Kidney	0.7814 ± 0.0552	0.796 ± 0.0295	0.762 ± 0.0251	0.804 ± 0.0334	0.982 ± 0.0676*
Lung	0.823 ± 0.0764	0.951 ± 0.0689	0.826 ± 0.0634	0.940 ± 0.1161	1.141 ± 0.1247
Colon	0.809 ± 0.0659	0.934 ± 0.0719	0.859 ± 0.0674	0.856 ± 0.0893	1.048 ± 0.1128

Values are expressed as means ± SEM.

* Significant different as compared to normal control, *p* < 0.05.

### Effect of MEMC_L_ on the incidence of ACF formation in AOM-treated colon of rats

The effect of MEMC_L_, at all tested doses, against AOM-induced ACF formation in carcinogenic rats is summarized in Table 2. All rats in the cancer group (Group 2) that received (AOM +8% Tween 80) demonstrated a 100% incidence of ACF formation whereas no ACF was detected in the normal group (Group I) that received (normal saline +8% Tween 80). There was also significant reduction in the number of ACF formed when comparison was made between the 500 mg/kg of MEMC_L_-treated group against the cancer group (Group 2). Higher number of ACF was observed in the distal part of colon for the cancer group and MEMC_L_-treated groups. The reduction in percentage of ACF formation recorded for the MEMC_L_-treated groups in comparison with the cancer group ranged between 21% and 55% (Figure 2).

**Table 2.** The incidence of aberrant crypt foci (ACF) found in rats colon induced by azoxymethane (AOM).

				Incidence of ACF		Reduction as compared
Group	Treatment	Proximal	Middle	Distal	Total of ACF	to AOM control (%)
I	Normal control	0.00 ± 0.00	0.00 ± 0.00	0.00 ± 0.00	0.00 ± 0.00	
II	AOM control	33.00 ± 3.055	27.0 0 ± 2.887	47.67 ± 3.480	107.7 ± 6.489	
III	AOM + 50 mg/kg MEMC	24.67 ± 1.333*	27.33 ± 4.910	33.33 ± 5.696	85.33 ± 9.563	20.77%
IV	AOM + 250 mg/kg MEMC	23.0 ± 0.577**	25.0 ± 2.517	28.0 ± 3.512*	76.0 ± 6.000	29.43%
V	AOM + 500 mg/kg MEMC	14.33 ± 1.453***	19.67 ± 1.667	23.00 ± 4.509**	48.33 ± 13.48**	55.13%

Values are expressed as means ± S.E.M.

* Significant different as compared to AOM control, *p* < 0.05.

** Significant different as compared to AOM control, *p* < 0.01.

*** Significant different as compared to AOM control, *p* < 0.001.

### Effect of MEMC_L_ on antioxidant activities of AOM-treated colon tissue homogenate

Table 3 shows the level of SOD, CAT, GSH and MDA in AOM-treated colon following treatment with MEMC_L_. There was a significant (*p* < 0.05) decrease in the SOD level in Group 2 (cancer group) due to the action of AOM alone. However, pretreatment with MEMC_L_, at all doses, significantly (*p* < 0.05) reversed the AOM action on SOD level and increased the enzyme's level towards the normal value.

**Table 3.** Effect of antioxidant enzymes in the colons of rats subjected to MEMC treatment.

			Antioxidant assays		
Group	Treatment	SOD (U/mg protein)	CAT (nmol/min/mL)	GSH (µM/mg protein)	MDA (ng/g tissue)
AOM control	AOM	0.6473 ± 0.2107	95.33 ± 1.202	16.55 ± 1.913	3.097 ± 0.2621
Normal control	Normal saline	2.672 ± 0.1521***	103.4 ± 0.1764	39.57 ± 4.534***	1.520 ± 0.2403**
Treatment	AOM + 50 mg/kg MEMC	2.104 ± 0.0610***	101.9 ± 0.03333	23.13 ± 0.777*	2.460 ± 0.2879*
Treatment	AOM + 250 mg/kg MEMC	2.352 ± 0.0706***	102.7 ± 0.0*	25.41 ± 1.150*	2.343 ± 0.2418*
Treatment	AOM + 500 mg/kg MEMC	2.526 ± 0.0878***	108.1 ± 4.942**	32.06 ± 1.782**	1.200 ± 0.1015***

Values are expressed as means ± S.E.M.

* Significant different as compared to AOM control, *p* < 0.05.

** Significant different as compared to AOM control, *p* < 0.01.

*** Significant different as compared to AOM control, *p* < 0.001.

The amount of CAT activity in the cancer group (Group 2) was also significantly (*p* < 0.05) reduced in comparison with the normal group (Group 1). Treatment with MEMC_L_, at 500 mg/kg, caused significant (*p* < 0.05) elevation in the level of CAT activity in comparison with the cancer group (Group 2).

The level of GSH was significantly (*p* < 0.05) depleted in cancer group (Group 2) when compared with the normal group (group 1). MEMC_L_ treatment increased the GSH level when compared with the cancer group.

As seen in Table 3, the level of MDA was significantly (*p* < 0.05) elevated in the cancer group when compared with the normal group. Treatment with MEMC_L_ caused significant (*p* < 0.05) reduction in the MDA level towards the normal value in a dose-dependent manner.

### Identification of flavonoid in MEMC_L_ via HPLC analysis

Figure 3 shows the HPLC profile of MEMC_L_ wherein five major peaks were detected. However, comparison with the respective chromatogram of several pure flavonoids mentioned earlier at 366 nm demonstrated that only rutin peak matched peak number 2 in the chromatogram of MEMC_L_.

Figure 3.Comparison between chromatogram of the standard compound (A) rutin and (B) quercitrin with the chromatogram of MEMC at 366 nm showing that the peak of rutin was parallel to one of the peaks presence in MEMC.

**Figure:**
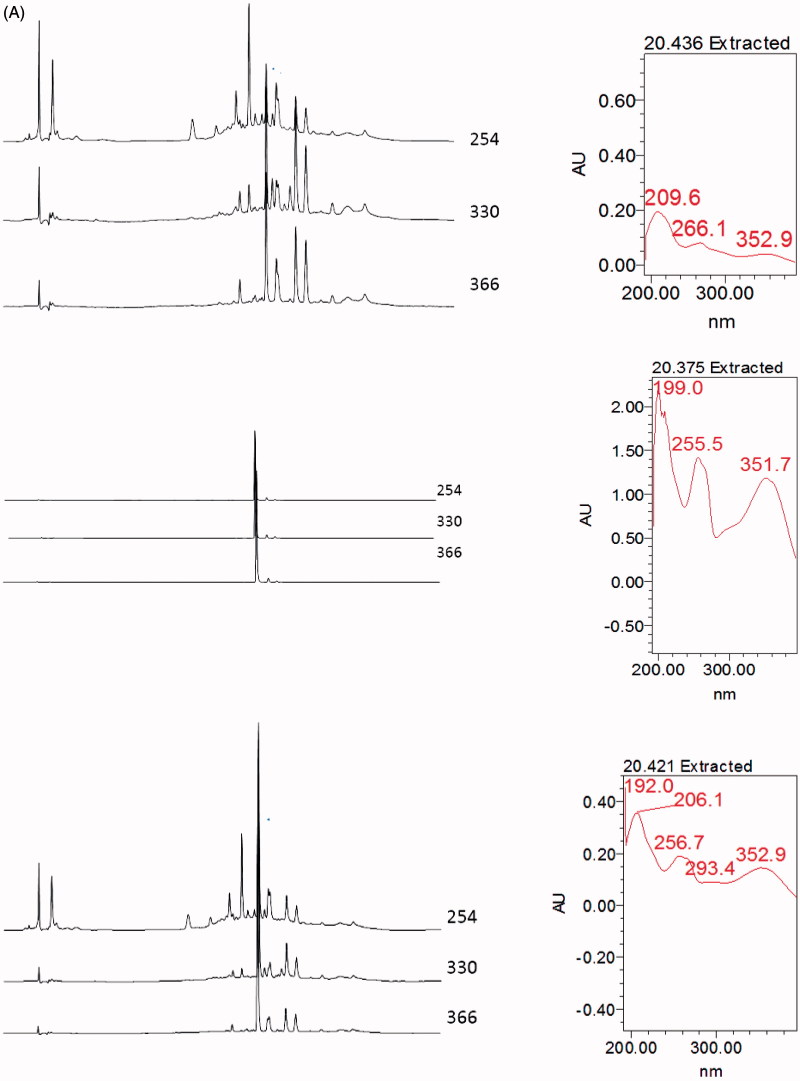

**Figure:**
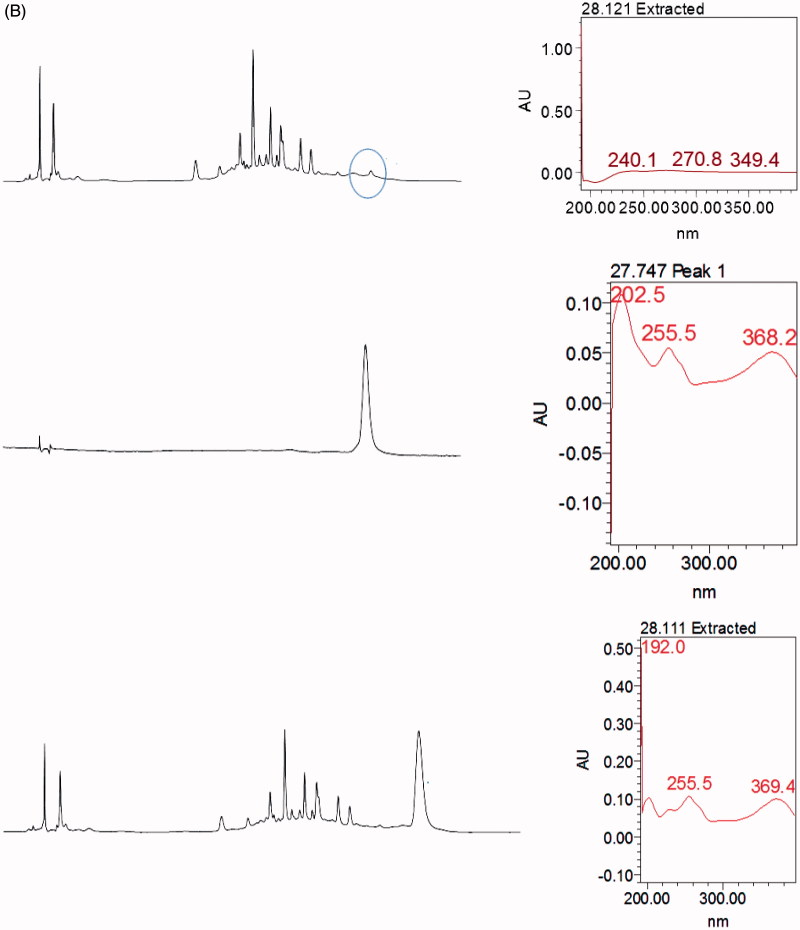

### Identification of phenolic compounds in MEMC_L_ using the UHPLC–ESI-MS

**Figure 4.**
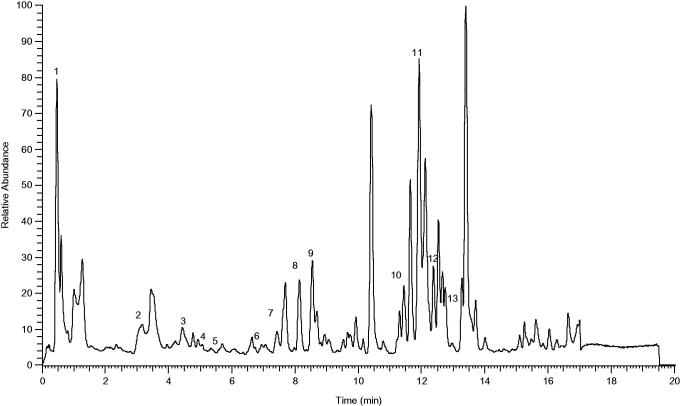
Total ion chromatography (TIC) of MEMC obtained using the UHPLC instrument in negative ion mode.

The phenolic compounds profile of MEMC_L_ obtained via the UHPLC-ESI-MS analysis is shown in Figure 4. The chromatogram obtained following the analysis shows the presence of various peaks of which 13 peaks were identified as listed in Table 4.

**Table 4.** Phenolic compounds identified in MMCL by UHPLC-MS.

Peak no.	*t*^R^ (min)	[M–H]–	Error (ppm)	Formula	Identification
1.	0.46	169.01392	5.090	C_7_H_5_O_5_	Gallic acid
2.	3.16	193.05023	3.599	C_10_H_9_O_4_	Ferulic acid
3.	4.58	599.10559	4.079	C_28_H_23_O_15_	Quercitrin-2″-*O*-gallate
4.	5.08	447.09439	4.926	C_21_H_19_O_11_	Kaempferol-3-*O*-galactoside
5.	5.39	463.08957	5.392	C_15_H_9_O_8_	Quercetin-3-*O*-galactoside
6.	6.96	583.11093	4.576	C_28_H_23_O_14_	Afzelin-*O*-gallate
7.	7.44	603.07947	4.209	C_30_H_19_O_14_	Quercetin dimer
8.	8.12	593.13110	3.596	C_30_H_25_O_13_	Kaempferol-3-*O*-glucoside
9.	8.57	271.06100	3.321	C_15_H_12_O_5_	Pinobaksin
10.	11.45	255.06644	4.919	C_15_H_11_O_4_	Pinocembrin (isomer 2)
11.	11.92	299.05606	3.496	C_16_H_11_O_6_	Kaempferide I
12.	12.39	299.05649	4.934	C_16_H_11_O_6_	Kaempferide II
13.	12.78	313.07214	4.713	C_17_H_13_O_6_	Ermanin II

## Discussion

The present study for the first time reported on the anti-colon cancer potential of MEMC_L_ against AOM-induced colon cancer in rats model hence confirmed the previous claims on the association between the antioxidant and anti-inflammatory activities as a part of the mechanisms of anticancer activity (Henley 2002; Devasagayam et al. 2004; Rayburn et al. 2009; Waly et al. 2012). Besides, this study further had proven the presence of anti-colon cancer activity in MEMC_L_ via an *in vivo* model, which supported earlier findings using the *in vitro* model (Mahmood et al. 2014). In addition, the present study also confirmed on the ability of MEMC_L_ to modulate the endogenous antioxidant system consisting of SOD, CAT, GSH and MDA as a part of the anticarcinogenesis mechanisms against the action of AOM (Waly et al. 2012). Furthermore, MEMC_L_ feeding did not affect the body weight gain profiles, which is consistent with previous anti-cancer efficacy studies using the same cancer models (Derry et al. 2013).

AOM can trigger oxidative and DNA damage following its administration due to its highly reactive metabolite that is methyldiazonium ion, which can trigger mutagenicity by initiating chromosomal damage and induction of micronuclei (MN) cells, results in colonic morphological changes associated with aberrant crypt foci (ACF) development. ACF are putative preneoplastic lesions that appear as abnormally large, darkly stained and slightly elevated from the normal crypt, display an irregular glandular architecture at epithelial region and have possibility to develop into colorectal cancer. Normally, it can be seen as early as 2–4 weeks after carcinogen administration (Caderni et al. 1995). The ability of MEMC_L_ to reduce the ACF count might also be attributed partly to the extract high antioxidants content and ability to modulate the endogenous antioxidant system within the colon tissue.

One of the potent intracellular antioxidants to eliminate the oxidative stress is GSH. The antioxidant capability of GSH is achieved by direct interaction of the –SH group with ROS or work as coenzyme to detoxify the ROS reaction (Hamiza et al. 2012). As described earlier, AOM induces oxidative stress by depleting the level of GSH; in the present study treatment with MEMC_L_ restores the GSH level indicating the protective effect of MEMC_L_. Besides GSH, AOM also depleted the level of CAT and SOD, which was reverse and restore back towards normal value by MEMC_L_. The administration of AOM also induced a marked increase in MDA level, which was reduced by MEMC_L_ indicating the cytoprotective action of the extract.

Following the administration of AOM, marked inflammation was observed in the colonic tissue surrounding the ACF area suggesting that the AOM-induced oxidative stress was partly mediated by an inflammatory response. It is well acknowledged that the inflammatory process was a part of the cells natural defence against tissue damage, which was generally associated with oxidative stress (Henley 2002; Rayburn et al. 2009). The presence of inflammatory cells in the AOM-treated group was remarkably attenuated by the MEMC, as the plant reported previously to have anti-inflammatory properties (Mahmood et al. 2014).

In the present study, rutin was detected in MEMC_L_ via the HPLC analysis at 366 nm. This compound has been reported to exert antioxidant, anti-inflammatory activities (Gautam et al. 2016) and anti-colon cancer activity (Volate et al. 2005). Rutin has been shown to target different modulators of Wnt signalling intracellularly (Perk et al. 2014), this action perhaps can cause negative modulation of APC gene (adenomatous polyposis coli). *APC* is one of the genes that directly involves in carcinogenesis of colon cancer, thus preventing the *APC* gene forming its complex transcriptional factor might inhibit the early stage of colon cancer by *M. calabura* leaves.

Further subjection of MEMC_L_ to the UHPLC-ESI-MS analysis demonstrated the presence of various polyphenolic compounds as indicated by the presence of a range of peaks. Of these, 13 peaks were identified to represent the respective bioactive compounds (Table 4) and, interestingly, some of these compounds, such as gallic acid, ferulic acid and pinocembrin, have been reported to exert anticarcinogenic activity. Gallic acid exerts an anticarcinogenic activity against 1,2-dimethylhydrazine (DMH) induced colon carcinogenesis in rats via modulation of the endogenous antioxidant defence system (Giftson et al. 2010). Furthermore, gallic acid, the active component of grape seed procyanidins, has also been reported to show antiproliferative and antiapoptotic activities against pancreatic cancer cells (Cedó et al. 2014). In addition, gallic acid was also proven to exert a radical scavenging ability toward OH and OOH radicals (Marino et al. 2014). Ferulic acid has also been reported to exert an anticarcinogenic activity against azoxymethane-induced colon carcinogenesis (Kawabata et al. 2000) possibly via the reduction of proliferative activity and the induction of apoptosis in cancer cells (Srinivasan et al. 2007). Moreover, Srinivasan et al. (2007) also cited the ability of ferulic acid to exert antioxidant and anti-inflammatory activities, which are generally known to play part in the mechanism of anticancer. Pinocembrin has also been cited to possess antioxidant, anti-inflammatory and anticancer activities (Santos et al. 1998; Liu et al. 2008; Rasul et al. 2013). Based on these reports, it is plausible to suggest that MEMC_L_ exerts an anticarcinogenic activity against AOM-induced colon cancer in rats partly through the synergistic action of these chemicals.

## Conclusions

In conclusion, the results of this study suggested that MEMC_L_ has a potential effect against the AOM induced colonic preneoplastic progression in Sprague–Dawley rats. Besides, this study also demonstrated the administration of MEMC_L_ attenuates the AOM-induced alterations at lipid peroxidation level and in the overall antioxidants enzymatic status of the rat′s colon. MEMC_L_ may have potential as chemoprevention agent possibly by reducing the colonic oxidative stress, increasing the antioxidants levels possibly via the synergistic action of several flavonoids, including rutin, gallic acid, ferulic acid and pinocembrin.
